# Evaluating the impacts of grades on vehicular speeds on interstate highways

**DOI:** 10.1371/journal.pone.0184142

**Published:** 2017-09-01

**Authors:** Xinqiang Chen, Zhibin Li, Yinhai Wang, Zhiyong Cui, Chaojian Shi, Huafeng Wu

**Affiliations:** 1 Merchant Marine College, Shanghai Maritime University, Shanghai, China; 2 Department of Civil and Environmental Engineering, University of Washington, Seattle, United States of America; 3 School of Transportation, Southeast University, Nanjing, Jiangsu Province, China; 4 Transportation Data Science Research Center, College of Transportation Engineering, Tongji University, Shanghai, China; Beihang University, CHINA

## Abstract

Grade variation on interstate highways affects the roadway geometric design, vehicle performance and driver behavior, thus possibly exerting an unexpected effect on vehicular speed. Hence, determining the internal relationship between grade and speed is important and useful for drivers, traffic regulators and other traffic participants. However, the problem with performing this research is the lack of large-scale gradient and speed data. Google Earth (GE) provides an application programming interface for extracting elevation data worldwide. The elevation dataset from GE can be easily converted to grade data. In addition, our team has collected and stored speed series data for different freeways over several years. Based on the above obtainable grade and speed datasets, we conducted research on the effect of grades on free flow speeds from two perspectives. First, the influence of grades on speed was analyzed from both quantitative and qualitative aspects. The analysis of the distributions of four typical types of speeds demonstrated a decreasing tendency as the speed increased. Steeper grades generated a more intense speed fluctuation in terms of the four types of speeds. Second, a model based on the Student’s t-test was developed to evaluate the level of significant difference among speed series under neighboring grades. The Student’s t-test demonstrated that adjacent grades do not significantly influence the speeds. In summary, speeds under different grades showed obviously different tendencies. The findings of this study can help transport authorities set more reasonable speed limits and improve the geometric design of interstates with grade variation constraints.

## Introduction

In transportation, the roadway gradient is a critical factor, in addition to fuel consumption analysis, traffic congestion prediction, velocity design [1], highway capacity analysis, and emergency evacuation planning. Previous studies showed that vehicular speed is significantly affected by grade variation. Silvas et al. reported that gradient variation constrains the vehicle’s acceleration and deceleration performance, especially for heavy vehicles [2]. Sentouh presented a method of obtaining the maximum speed under different grades considering the influences of pedestrian behavior in traffic, vehicle performance and road conditions [3].

Many researchers have concentrated on the effect of the freeway on urban roadway speed. Boriboonsomsin and Barth presented a method to determine the optimal speed using the variation of fuel efficiency under different grades [4]. In addition, speed and automobile horsepower were employed to estimate the road gradient and other traffic parameters [5]. Ma et al. performed studies on predicting traffic speed and traffic jam conditions under different roadway grades [6, 7]. Tang et al. employed a speed-gradient-based model to initialize, evaluate and predict traffic flow-related parameters [8–13]. However, several factors in addition to grade resulted in speed variation on downtown roadways.

Studies were performed under the expressway conditions to further determine the relationship between speed and grade. Li et al. investigated the effect of slope on traffic flow and speed for single-line motorways [14]. Mathematical models were proposed for optimizing the highway gradient design, considering both the cost of road construction and the designed speed [15]. Zhou et al. introduced a speed prediction model based on different grades under highway free flow conditions [16]. After exploring the intrinsic relationship between the elements of grade-relevant factors and traffic velocity, Xu et al. constructed a speed prediction model for different grades and different vehicle types [17]. Although these studies focused on the relationship between slope and speed for the expressway, less attention was focused on the effect of speed and slope under free flow conditions.

However, the free flow condition is the most common situation that drivers encounter when they are driving on the freeway. In addition, a more accurate relationship between speed and grade can be obtained under free flow conditions because no other interferences (e.g., traffic jams and traffic lights) exist. Hence, exploring the intrinsic relationship between slope and velocity under free flow conditions is urgently required. Accurate gradient and speed data under free flow conditions are necessary for conducting this research. However, no large-scale grade and speed data are accessible to the public. Our team has performed many studies to obtain and store grade- and speed-related data [18, 19].

Based on the available large-scale datasets for grade and speed, we focus on the effect of grades on speed under free flow conditions. Our primary objective is to determine the relationship between different road gradients and velocity on the freeways. First, we analyzed the distribution of typical types of speeds under different grades from both quantitative and qualitative aspects. Second, we used the Student’s t-test (t-Test) to determine the significance level of speeds under different grades. The t-Test results demonstrated that the speeds at different grades are significantly different. The remainder of the paper is organized as follows. The following section introduces the data source and models used for the research. Then, the next section analyzes the influence of grades on speed. Finally, we conclude our paper and briefly describe future studies.

## Data source and method

### Grade data

Currently, the freeway grade information cannot be directly accessed by the public. However, Google Earth (GE) provides elevation data, which are indirect grade data. GE records elevation worldwide, and the elevation data are extractable from GE’s application programming interface (API) [18]. However, some typical types of outliers have been found in GE’s raw elevation data. Hence, we employed the ensemble empirical mode decomposition (EEMD) method to smooth out the anomalies in the elevation dataset [20]. The EEMD model decomposes the initial noisy elevation data into several intrinsic mode functions (IMFs). The energy level of noisy IMFs was less than zero, while that of the useful IMFs was larger than zero. Thus, an energy-based mechanism was developed to discriminate the useful IMFs from the noisy IMFs. Additionally, we compared the gradient data obtained from EEMD-smoothed elevation data with the Highway Safety Information System (HSIS) gradient data. We found that the EEMD-based gradient data were consistent with the HSIS data. Hence, the EEMD-based grade data are a reliable grade source. To ensure that continuous and reasonable grade data are obtained, the criterion from the American Association of State Highway and Transportation Officials’ (AASHTO) “A Policy on Geometric Design of Highways and Streets” is used to exclude grade outliers [21].

According to the criterion from AASHTO, the maximum grade primarily relies on the design speed and the freeway terrain. In general, 6% is the steepest grade that is acceptable for most freeways in the United States. A road gradient of 7% is only allowed on some extremely steep freeway segments. Hence, a grade value higher than 6% is considered a potential outlier. For the potential abnormal freeway grades, our team manually checked the terrain from the GE street map to determine the normal and abnormal grade points. For a given freeway segment h, its grade G(h) is calculated using Eq (1):

$$G(h)=\frac{E_{beg}(h)-E_{end}(h)}{M_{beg}(h)-M_{end}(h)}$$ (1)

where E_*beg*_(h) is the elevation at the beginning point of segment h, while E_*end*_(*h*) is the elevation at the ending point. The parameters of M_*beg*_(*h*) and M_*end*_(*h*) are the beginning and ending mileposts of segment *h*, respectively.

### Speed data

Interstate 5 and Interstate 90 are the busiest and longest expressways, respectively, in Seattle, Washington (WA). In addition, Interstate 90 has more grade changes than those on other freeways in WA. Hence, we explore the relationship between slope and velocity based on the speed data obtained from Interstate 5 and Interstate 90. Our team developed speed-related databases that store various types of speeds. To reduce the influence from random factors, such as weather, temporary traffic control, and road construction, several measurements are obtained and prerequisites are assumed:

1. Speed must be sampled under relatively good weather conditions at the sites. Rainy season in Seattle occurs from November to February. Hence, the weather’s influence cannot be ignored when the relationship between speed and grade is analyzed during this period. However, it is always sunny from March to October in Seattle; therefore, the speed data were collected during this period, considering the weather conditions.
2. To determine the speed and grade relationship under free flow conditions, we discarded speed data during the peak-hour periods on weekdays. In addition, to avoid the situation in which only a few vehicles pass the detectors, the timestamp was not be too late or too early in the day. Our team have carefully determined the appropriate timestamps by manually checking the traffic volume data on the DriveNet, which is a transportation big-data platform developed by researchers in University of Washington [19, 22]. The data check showed that the traffic volume from 00:00 AM to 3:00 AM was significantly smaller than the volume during other time intervals. The traffic volume recorded in our database confirmed the above analysis. Hence, the timestamps for collecting the speed data included the following: 3:00 AM to 7:00 AM, 9:00 AM to 5:00 PM and 7:00 PM to 00:00 AM.
3. People are likely to travel or sightsee during National holidays. Thus, the speed distribution may show unusual patterns on National holidays. Additionally, the speed data stored in our databases showed an obviously different variation tendency on holidays, such as Independence Day, Labor Day, Columbus Day and so on. Hence, we also discarded the speed data during festivals and holidays to exclude the holiday interference.

The above conditions can provide reliable speed data series. However, we developed a data quality control process to further improve the quality of the speed series. The speed data that are collected during temporary traffic control periods, such as roadway construction and sports events, show obvious and different variation tendencies. Our data quality control process can detect and label abnormal speeds automatically. For instance, when a segment of the roadway is under construction, the speed from the site will stay at 0 miles/h for several days. Hence, the data quality control process will estimate the speed based on the historical normal speed records at the site. Thus, we can obtain reliable speed series under different grades using the above-mentioned measurements.

### Method

Statistical methods are employed to determine whether neighboring grade speeds are significantly different from each other. Note that neighboring grades indicate that the values of a segment’s grade are adjacent to each other. Given an upslope segment with a grade of 0.03, its neighboring grades are 0.02 and 0.04. It is not essential that the neighboring grades are collected from the neighboring freeway segments in our study.

Here, we employed the t-Test model to verify the neighboring grade’s effect on speed. The t-Test method primarily involves a one-sample t-Test, a two-sample t-Test and a paired-samples t-Test. The data series for the one-sample t-Test are acquired from the same population. However, the data sequences for the two-sample t-Test and paired-samples t-Test are obtained from two populations. The one-sample t-Test is the basic statistical method for the t-Test family. The two-sample t-Test and paired-samples t-Test are considered as derivatives of the one-sample t-Test. Hence, we describe the one-sample t-Test in detail first. Then, we demonstrate the work flow of the two-sample t-Test for evaluating the effect of the neighboring grades on speed in detail.

The first step of the one-sample t-Test is to develop a null hypothesis and an alternative hypothesis. Suppose the null hypothesis *H*_*n*_ and alternative hypothesis *H*_1_ are listed in Eqs (2) and (3), respectively. Then, the t-statistics calculation is employed to determine whether to accept or reject the null hypothesis. The calculation of *t*_*sta*_ (t-statistics) is shown in Eq (4). We employ the significance level to demonstrate the probability of accepting or rejecting the null hypothesis. The significance level is labeled with the symbol *α*. We will accept the null hypothesis if *t*_*sta*_ < *t*_*α*_ where *t*_*α*_ is the threshold for the t-statistics:

$$H_{n}:\;{\bar{V}}_{{grade}_{i}}=\mu_{0}$$ (2)

$$H_{1}:\;{\bar{V}}_{{grade}_{i}}\neq \mu_{0}$$ (3)

tsta=DifVarnum (4)

where ${\bar{V}}_{{grade}_{i}}$ is the average speed for the grade *i* and *μ*_0_ is the expected speed at grade *i*. The *t*_*sta*_ is the t-statistics and *Dif* is the difference of ${\bar{V}}_{{grade}_{i}}$ and *μ*_0_. The parameter *Var* is the variance for the speed series under grade *i*. The *num* is the size of the speed series under grade *i*.

Intuitively, the speed at a steeper uphill segment is more likely to obtain lower values. In addition, a steeper downhill segment results in higher speed values. Based on the above analysis, we propose the null hypothesis and alternative hypothesis for the two-sample t-Test as shown in Eqs (5) and (6), respectively. The null hypothesis used in the two-sample t-Test is that the speed series are insignificantly different from each other under neighboring grades. Thus, we assume that the neighboring grades significantly affected the speed data. The parameters *α* and *t*_*α*_ are the significance level and the t-statistics threshold, respectively, which were previously discussed.

The T(t) in Eq (7) is the value of the t-Test that decides whether to accept or reject the null hypothesis. Specifically, speeds under neighboring grades are insignificantly different when the value of T(t) is smaller than *t*_*α*_. Therefore, we will accept the null hypothesis if T(t) < *t*_*α*_. Otherwise, a small probability event occurs when the value of T(t) is larger than the *t*_*α*_. Thus, speeds under neighboring grades are significantly different. Therefore, we reject the null hypothesis if T(t) > *t*_*α*_:

$$H_{n}:\;{\bar{V}}_{{grade}_{1}}={\bar{V}}_{grade_{2}}$$ (5)

$$H_{1}:\;{\bar{V}}_{{grade}_{1}}>{\bar{V}}_{grade_{2}}$$ (6)

$$T(t)=\frac{\left|{\bar{V}}_{{grade}_{1}}-{\bar{V}}_{grade_{2}}\right|}{\sqrt{\frac{s_{1}^{2}}{n_{1}}+\frac{s_{2}^{2}}{n_{2}}}}$$ (7)

where T(t) is the value of t-statistics and ${\bar{V}}_{{grade}_{i}}(i=1,2)$ is the average speed for the grade *i*. Parameter *S*_*i*_(*i* = 1,2) is the standard speed deviation for the corresponding grade. Parameter *n*_*i*_(*i* = 1,2) is the length of the sampled speed series.

## Results and analysis

Considering the roadway geometry characteristics in Seattle, we extracted the grade and speed data using the mileposts between 230.14 km (143 miles) and 305.78 km (190 miles) for Interstate 5. The speed and grade data on Interstate 90 were obtained at the mileposts from 4.02 km (2.5 miles) to 27.36 km (17 miles). Negative grade data indicate that the segment is a downhill slope, and the corresponding gradient value is the absolute value of the data. Positive grade data indicate an uphill segment, and a value of 0.00 demonstrates a flat freeway section.

The obtained grade data on Interstate 5 mainly range from -0.04 to 0.04 with a grade interval of 0.01. A grade of -0.04 for a segment means that the segment is a downhill slope, and its absolute gradient is 0.04. Similarly, a grade of 0.04 is an uphill segment, and the absolute gradient value is 0.04. There are no sufficient speed data at grade 0.04 for Interstate 90; thus, the typical grades for Interstate 90 range from -0.03 to 0.03. In the following sections, we evaluated the effect of grades on the speed series at different interstate segments. Then, the t-Test method was developed to verify the effectiveness of the relationship between grade and speed.

### Impacts of grades on vehicular speed

We used four typical types of velocities to demonstrate the relationship between grade and speed. The four types of speeds involved the maximum, average, 85% and 15% speeds. The maximum speed (Max) is the maximum speed for different grades in the sampling speed series. We used the term “Mean” to represent the average speed under different grades. A value that is 85% of the speed series is labeled V85%. Similarly, the symbol V15% represents a value that is 15% of the speed for different grades in the speed series.

These four types of speeds played important roles in evaluating freeway safety, operation efficiency, and the design speed. A higher maximum speed leads to a higher road occupancy and better traffic efficiency. In addition, the maximum speed is more sensitive to the grade variation. The average speed provides the overall variation trend of the speed series. The fluctuation of the average speed demonstrated the grade’s effect on the whole speed series. In addition, the V85% and V15% speeds are the major indicators for the freeway speed design of different freeway classes. In addition, the standard deviation (SD) and variance (Var) demonstrated the speed fluctuations under different grades. These two indicators demonstrated the grade’s influence on speed from a statistical viewpoint. Based on the above analysis, it is reasonable to employ the Max, Mean, V85%, and V15% speeds as well as the two statistical indicators (SD and Var) to explore the internal relationship between speed and grade.

Generally, a downhill slope will accelerate a vehicle’s speed while an uphill segment will decelerate the speed. A steeper grade section results in a more obvious influence on vehicular speed in terms of acceleration or deceleration. The distribution of the speed indicators for both the Interstate 5 and Interstate 90 segments under different grades is shown in Table 1 and Table 2 and confirms the above perceptions. The Max column reveals that the speed under grade -0.04 is higher than that under other grades. The maximum speed for grade -0.04 is 118.82 km/h. The speed of grade -0.03 is almost the same as that of grade -0.04. However, the Max speed under grade -0.02 is 8% lower than the speed at gradient -0.04. The speed variation between -0.04 and 0.04 showed a significant decreasing tendency in the Max speed column.

**Table 1:** Speed indicators for the Interstate 5 segment.

Grade	Max (km/h)	Mean (km/h)	V85% (km/h)	V15% (km/h)	SD	Var
-0.04	118.82	111.20	115.38	107.96	3.87	14.98
-0.03	117.28	107.37	112.49	103.17	4.67	21.76
-0.02	108.86	102.04	105.78	98.95	5.64	31.77
-0.01	107.22	99.66	103.12	96.3	3.09	9.55
0.00	107.56	97.73	102.07	94.53	3.48	12.11
0.01	104.30	96.13	100.50	93.23	4.20	17.65
0.02	102.85	94.02	97.78	89.12	4.48	20.07
0.03	97.80	92.41	95.75	89.52	3.54	12.55
0.04	93.34	82.05	87.75	80.11	12.84	164.82

**Table 2:** Speed indicators for the Interstate 90 segment.

Grade	Max (km/h)	Mean (km/h)	V85% (km/h)	V15% (km/h)	SD	Var
-0.03	120.11	105.34	110.7	99.36	6.08	36.95
-0.02	116.96	102.38	107.43	97.59	5.43	29.43
-0.01	121.11	99.5	105.2	93.92	7.62	58.14
0.00	110.49	96.96	101.87	91.68	5.7	32.48
0.01	101.93	95.05	98.13	91.58	2.97	8.82
0.02	109.07	93.24	99.03	85.55	6.44	41.5
0.03	107.27	88.27	97.9	79.34	9.07	82.27

The Mean column in Table 1 showed similar speed variations under different grades. Here, the variation of the average speed revealed an internal relationship between speed and grade. The average speed diminished slightly as the grade increased. The maximal value in the Mean column for Interstate 5 is 111.2 km/h, which was obtained under grade -0.04. The speed under grades 0.03 and 0.04 experienced a significant decreasing tendency, as the speed for grade 0.03 is 92.41 km/h and that for grade 0.04 is 82.05 km/h. The speed distribution of the V85% series is similar to the average speed distribution. The V85% speeds show a decreasing trend from grade -0.04 to 0.03. However, an obvious decrease for the V85% speed series was found between grades 0.03 and 0.04.

**Fig 1:**
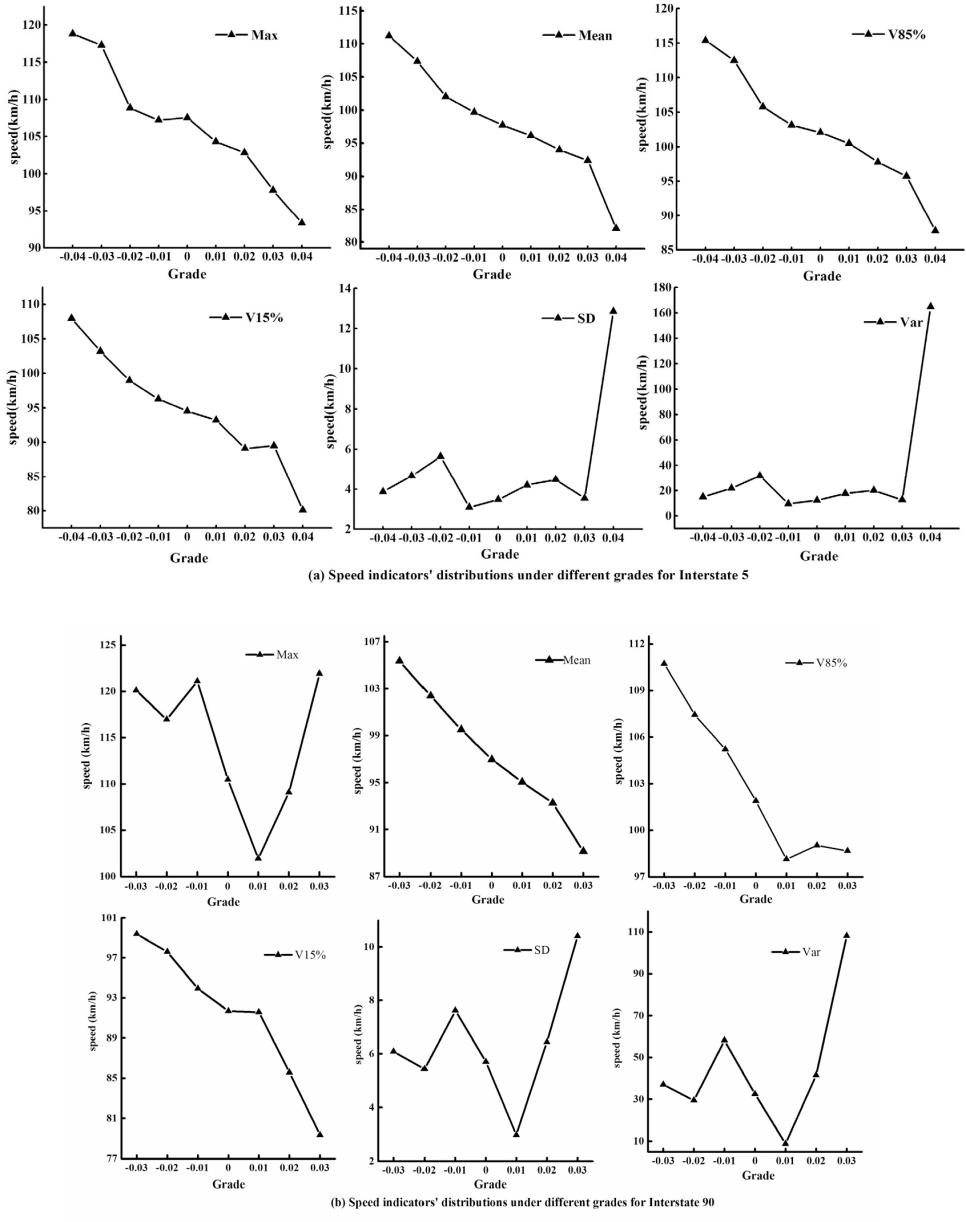
Speed indicator distributions under different grades for freeways: (a) Interstate 5 and (b) Interstate 90.

The V15% speed series showed a slightly different variation from that of V85% and the Mean speed. Specifically, the speeds at grades from -0.04 to 0.02 experienced roughly the same tendency as that at the V85% speed. However, the V15% speed under grade 0.03 mildly exceeded the velocity of gradient 0.02, which was different from the V85% speed series. In addition, the V15% speed of grade 0.04 was 80.11 km/h, which was 12% lower than that of grade 0.03. In general, the V15% series experienced a similar speed decreasing tendency to the other three speed series. An analysis of the speed series variation (Max, Mean, V85% and V15%) of Interstate 5 reveals that the speeds show a gently decreasing trend as the downslope grades increase. For the upslope segments of Interstate 5, the speed distribution shows a mildly decreasing tendency, and dramatic speed decrease is observed between grades 0.03 and 0.04. The speed distributions in Fig 1(A) confirmed this analysis.

The statistical indicators of SD and Var in Table 1 demonstrate that the speed fluctuation is influenced by different grades. The SD values for grades between -0.04 and 0.03 were less than 6. Meanwhile, the SD value for grade 0.04 was 12.84, which is two-fold higher than the values of other grades. The Var column in Table 1 reveals similar fluctuations to those in the SD column. This variation was further verified by the distributions of SD and Var in Fig 1(A). The variations of SD and Var revealed that upslope segments have a more remarkable influence on vehicular speeds. Speeds under grade 0.04 experienced a drastic variation, as its values for both SD and Var were much higher than those of the other grades. The distributions of the SD and Var indicators agreed with the analysis of the other speed indicators for the Interstate 5 segment.

Speeds of different grades for the Interstate 90 segment were also analyzed to explore the internal relationship between velocity and gradient. The Max, Mean, V85% and V15% columns for Interstate 90 in Table 2 reveal a similar decreasing tendency of speed variation to that of Interstate 5. The Max speed for grade -0.03 was 120.11 km/h, which was 2% higher than the speed under grade -0.02. However, the maximum speed of grade -0.01 was slightly higher than that of grade -0.03, which was 121.11 km/h. There were two primary reasons for the phenomenon. First, the sample size of the speed series may not be large enough for evaluating the Max speed. Second, some aggressive drivers were likely to sharply accelerate their vehicle when no cars were present in the surrounding lanes.

The Mean and V85% speeds, two common and widely used speed indicators, demonstrated an obvious declining tendency as the grade increased, as shown in Table 2. For the distribution of Mean speed for Interstate 90, the maximum speed was 110.7 km/h under grade -0.03, and the Mean speed decreased by 3% at each increasing grade interval. We obtain similar results based on the V85% and V15% distributions in Table 2. The maximum values for the SD and Var indicators in Table 2 were 9.07 and 82.27, respectively, which were both obtained at grade 0.03. The variation of SD and Var showed that a grade of 0.03 had a more significant influence on reducing the speed compared with the lower grades listed in Table 2. The distributions of each indicator for Interstate 90 are plotted in Fig 1(B).

**Fig 2:**
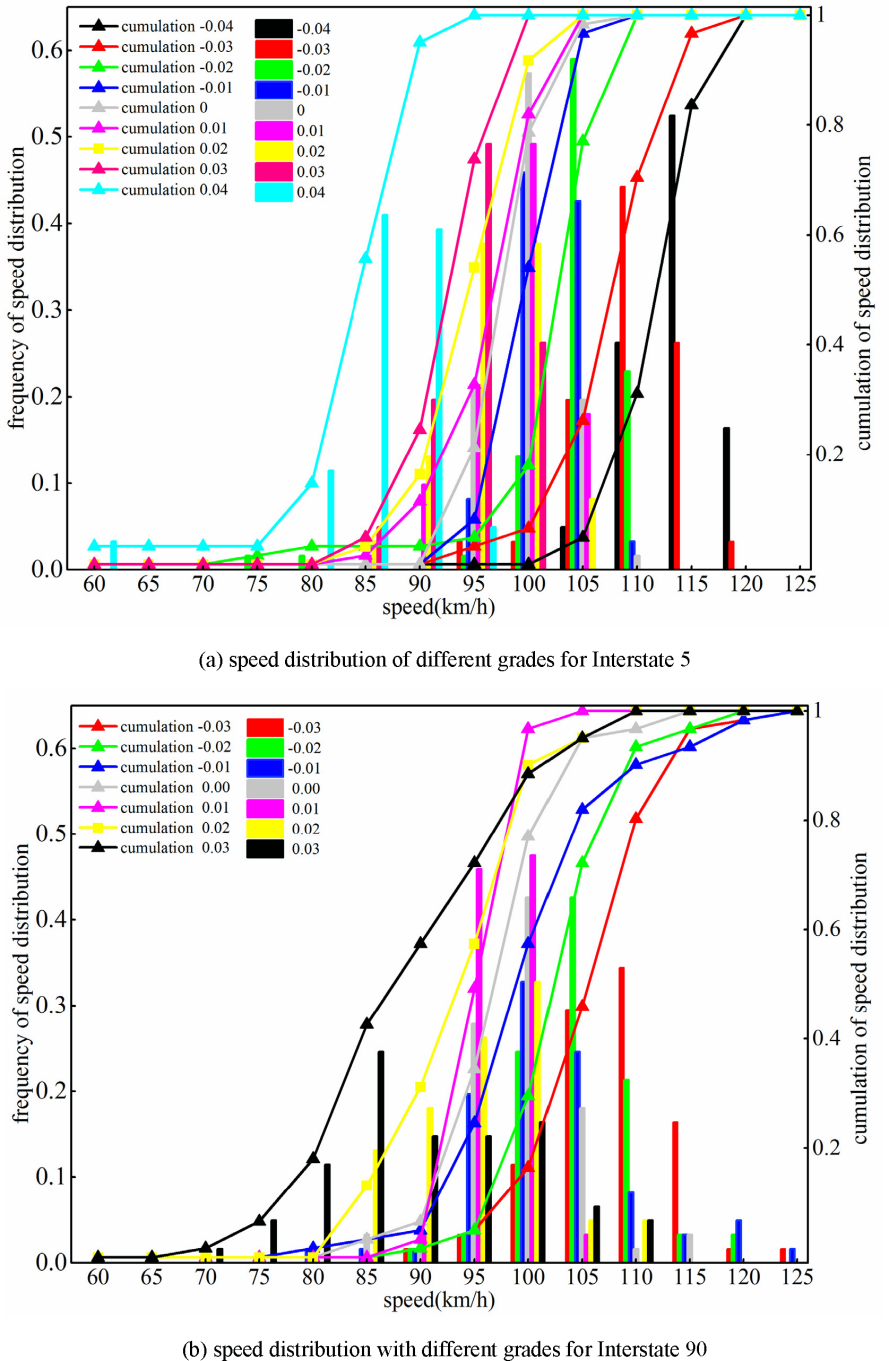
Speed distribution with different grades for the Interstate 5 and Interstate 90 segments.

We also plotted the speed distributions to explore the internal relationship between speed and grade. Fig 2 shows the speed distributions under different grades for the Interstate 5 and 90 segments. The primary y-axis is the speed frequency at different grades. The secondary y-axis is the accumulation of frequency for different grades. The speeds were divided into 14 groups, and each group was represented its maximal value. Specifically, each x-axis label represents an individual group. For instance, 60 km/h on the x-axis in Fig 2 represents speed group ranges from 55 km/h to 60 km/h. Similarly, 65 km/h in Fig 2 demonstrates speeds equal to or faster than 60 km/h and slower than 70 km/h. The other labels on the horizontal axis of Fig 2 have a similar meaning.

The black line and bar shown in Fig 2(A) shows that the speed under grade -0.04 is scattered over larger speed groups for Interstate 5. We noticed that almost 95% of the speed series under grade -0.04 was spread among the interval between 110 km/h and 125 km/h. The red line and bar in Fig 2(A) reveal that the speed under grade -0.03 is primarily centered over the interval between 105 km/h and 115 km/h. Almost half of the speed series at grade -0.03 ranges from 100 km/h to 110 km/h. For Interstate 5, the centroid of the speed distribution at a grade of -0.03 was much smaller than that at a grade of -0.04.

In fact, the centroid of the speed distribution showed a decreasing tendency as the gradient increased. The speed distribution at gradient 0.04 was mainly in the speed groups of 80 km/h, 85 km/h and 90 km/h, as shown by the cyan line and bar in Fig 2(A). The centroid of the speed distribution at grade 0.04 was much smaller than that at the other grades. This analysis demonstrates that larger grades decelerate the speed more obviously, thus verifying the above-mentioned analysis for the distributions of speed indicators (including Max, Mean, V85% and V15%) for the Interstate 5 segment.

The speed distribution for Interstate 90 is shown in Fig 2(B). The speed for grade -0.03 is primarily concentrated from 105 km/h to 115 km/h. However, less than 5% of the speeds under grade -0.03 is scattered over the interval from 120 km/h to 125 km/h. The green line and bar shown in the second subplot in Fig 2 show the speed distribution of grade -0.02. Note that 40% of speeds for grade -0.02 is situated at the speed group of 105 km/h. In addition, almost 20% of the speed series for grade -0.02 is located at 100 km/h and another 20% is at 110 km/h. The speed distribution implies that the average speed of grade -0.02 is slower than the speed of grade -0.03. This distribution is consistent with our above analysis of the Mean indicator for Interstate 90.

The blue line and bar in Fig 2(B) show the speed distribution under grade -0.01. The speed distribution reveals the variation of speed indicators in Table 2. It was observed that 2% of speed series under grade -0.01 is located at the speed group of 125 km/h, and 75% of the speed series under grade -0.02 is located at the speed interval from 95 km/h to 105 km/h. Hence, we can infer that the centroid of speeds under grade -0.02 was 100 km/h. In addition, the average speed of grade -0.01, shown in Table 2, was 99.5 km/h, while its centroid was 99.5 km/h. The speed series for the remaining grades showed that larger grades resulted in smaller speed centroids for Interstate 90. The major proportion of the speed series for grade 0.00 is located over the interval from 95 km/h to 105 km/h.

For the upslope of grade 0.01, over 90% speed data is centered over the interval of 95 km/h and 100 km/h. Similarly, the steeper upslope segment resulted in a sharper decreasing tendency of the centroid velocity. The speeds for the steepest uphill segment of Interstate 90 with grade 0.03 showed a significantly smaller speed centroid. Nearly 25% of the speed is located at the speed group of 85 km/h. In contrast to the other speed distributions, the speed distribution under grade 0.03 is scattered over several speed groups. Thus, a steeper uphill slope results in a larger speed variation. In fact, a steeper uphill slope results in confining the speed to a smaller speed interval. Similarly, steeper downhill grades result in higher speed distributions. Hence, we can conclude that a steeper gradient of an upslope reduces the speed, while a downslope increases a vehicle’s velocity.

### Significance test for speed series under different grades

Based on the geometry characteristics of freeways, grades transit between adjacent segments are smoothly. Thus, vehicle speed may be affected by neighboring grades. Thus, speed series under adjacent grades are not significantly different if they are affected by contiguous grades. Here, we employed the t-Test model to determine whether the neighboring grades significantly affected the speed series. Intuitively, the tendency of speed variation under different grades is monotonous. Hence, we employed the one-tailed t-Test method to evaluate the significant differences of the speed data. The null hypothesis is that a speed at a smaller grade equals that at a neighboring larger grade. We set the level of significance α as 0.05, which is an empirical value for many studies. The t-Test results for the different speed series of the Interstate 5 and Interstate 90 segments are shown in Table 3.

**Table 3:** The t-Test results of speed series under different grades.

	Grade set	t-Statistics	t-Critical	P-Value
**Interstate 5**	-0.04 VS -0.03	4.931	1.658	0.000
	-0.03 VS -0.02	5.690	1.658	0.000
	-0.02 VS -0.01	2.893	1.661	0.002
	-0.01 VS 0.00	3.236	1.658	0.001
	0.00 VS 0.01	2.299	1.658	0.012
	0.01 VS 0.02	2.685	1.658	0.004
	0.02 vs 0.03	2.200	1.658	0.015
	0.03 vs 0.04	6.075	1.667	0.000
**Interstate 90**	-0.03 VS -0.02	2.838	1.658	0.003
	-0.02 VS -0.01	2.406	1.659	0.009
	-0.01VS 0.00	2.083	1.659	0.020
	0.00 VS 0.01	2.317	1.662	0.011
	0.01 VS 0.02	1.993	1.663	0.025
	0.02 VS 0.03	3.490	1.660	0.000

The grade set column in Table 3 shows the speed data at the corresponding grades in the t-Test for comparison. Three variables were employed to demonstrate the t-Test results for the speed series at adjacent grades. The t-Statistics column list the t-Test values under the significance level of 0.05. The t-Statistics represent the value of T(t) in Eq (7). The significance values of the t-Test for each grade set are shown in the P-Value column in Table 3. The P-Value demonstrates the significance level of speeds under adjacent grades. The t-Critical column shows the critical value of the t-Test that serves as threshold. The t-Critical parameter is represented as *t*_*α*_. A detailed analysis of the t-Test for the grade-speed relationship of Interstate 5 and Interstate 90 segments is illustrated as follows.

For the Interstate 5 segment, the grade set of -0.04 vs. -0.03 showed a significant difference between the t-Statistics and t-Critical values. As shown in Table 3, the t-Statistics indicator for the grade set was 4.931, which was almost three-fold larger than the corresponding t-Critical value. In addition, the corresponding P-Value was 0.000, which was much smaller than the significant value of 0.050. All three indices confirmed that the speed data between -0.04 and -0.03 were obviously different. Hence, we rejected the null hypothesis and accepted the alternative hypothesis. Thus, the speeds under grade -0.03 were not affected by grade -0.04 and vice versa.

The t-Test results for the remaining grade sets of speed series for Interstate 5 showed a similar significant difference. The grade set of 0.03 vs. 0.04 showed the most significant difference between the t-Statistics and t-Critical values compared with the other grade sets. The smallest t-Statistics value was observed at grade set 0.02 vs. 0.03, which equaled 2.200. This value was 33% higher than the corresponding t-Critical value. Combining the distributions of t-Statistics and t-Critical indicators for Interstate 5, the speeds at adjacent grades were significantly different. Thus, it is reasonable to conclude that neighboring grades did not affect the speed series.

**Fig 3:**
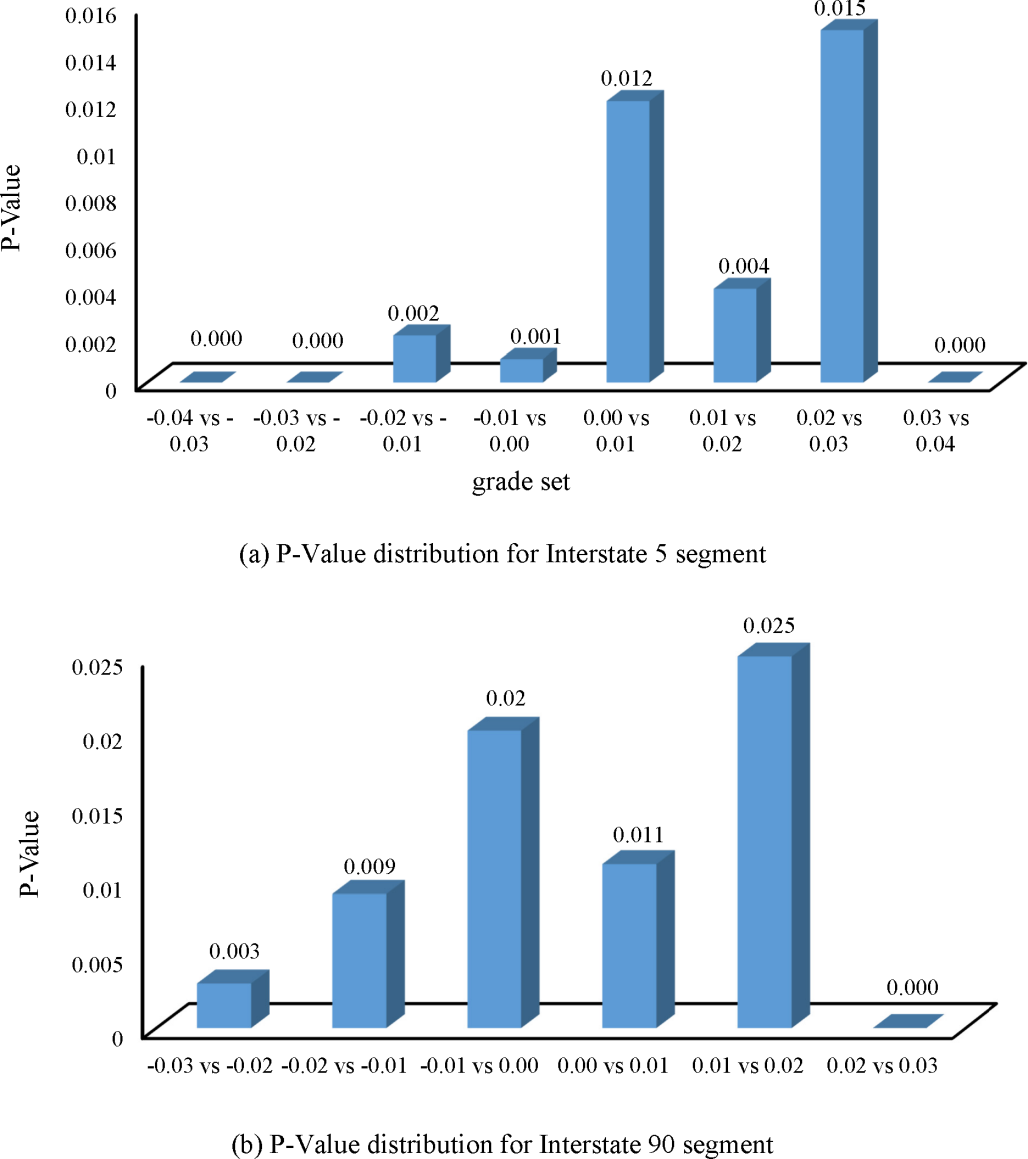
Significant test for different speed series of Interstate 5 and Interstate 90 segments.

The P-Value in Table 3 was the direct indicator demonstrating the grade effect on speed. A smaller P-Value showed a much larger difference between the two speed series for the grade set. As shown in Table 3, the minimum value for the P-Value for Interstate 5 was 0.000, which was obtained at grade sets of -0.04 vs. -0.03, -0.03 vs. -0.02, and 0.03 vs. 0.04. Moreover, the largest P-Value was 0.015, which was obtained from the grade set 0.02 vs. 0.03, demonstrating that speeds under grade 0.02 and 0.03 were more susceptible to each other’s grade compared with other grade sets. In addition, Fig 3(A) shows a P-Value distribution under different grade sets, which shows that the P-Values for Interstate 5 were smaller than the threshold of 0.05 for all the grade sets. Therefore, we conclude that adjacent grades do not significantly influence speed.

The statistical indicators for Interstate 90 showed a similar statistical performance to that of Interstate 5, as shown in Table 3. The largest value for the t-Statistics for Interstate 90 was 3.490, which was obtained from the grade set 0.02 vs. 0.03. The smallest value for t-Statistics was 1.993, obtained from the grade set of 0.01 vs. 0.02. All the t-Statistics values for Interstate 90 were larger than the corresponding t-Critical values in Table 3. In addition, the P-Values for Interstate 90 were significantly smaller than 0.05. The largest P-Value was 0.025, which was only half of the threshold P-Value. The second subplot in Fig 3 demonstrates the P-Value variation for Interstate 90.

The above analysis showed that the t-Test indicators do not support the null hypothesis for both Interstate 5 and Interstate 90. Specifically, all the t-Statistics indicator values were larger than the corresponding t-Critical values. Meanwhile, the P-Value indicators for both freeways were far less than the significant level of 0.05. Thus, we confidently can reject the null hypothesis. It is reasonable to conclude that neighboring grades do not significantly influence the speeds. The speed series were remarkably differently under adjacent grades for both Interstate 5 and Interstate 90.

## Conclusions

This study focuses on the relationship between speed and grade under free flow conditions for interstate highways. Based on the grades and speed data collected from GE and the speed database, we analyzed the speed distribution under different grades for the two typical interstates (Interstate 5 and Interstate 90) in Washington State. First, we studied the distributions of four typical speed series, including the maximal speed, average speed, 85% speed and 15% speed, under different grades. These four typical speeds showed an obvious decreasing tendency as the gradient increased. The variance and standard deviation for the four types of speeds demonstrated that upslope segments exert a more significant influence on speeds under free flow conditions. Second, we verified through the Student’s t-test measurement that speeds with neighboring grades were significantly different. Therefore, adjacent grades do not exert a significant influence on the speed.

In the future, some interesting studies can be performed based on the current findings. First, we can more accurately measure a vehicle’s fuel consumption under different grades and speed limits. Thus, drivers can drive at a more energy efficient speed on different freeway segments. Second, we can evaluate the combined influence from grade and speed on traffic volume under the free flow condition. Third, the findings of this study can be used to help traffic regulators design different speed limits under different grades more reasonably. Although our study found that grades significantly affected the corresponding speeds, and larger grades resulted in more intense speed variation. We did not determine which gradient was the most critical factor in accelerating or decelerating the speeds, which will be determined in further studies.

## Supporting information

S1 Dataset The dataset includes raw data for the speed distributions under different grades, student test results and speed indicators for both Interstate 5 and Interstate 90 freeways. (RAR)
